# CALPUCK: An Open Python Tool for Cremer–Pople Ring Puckering Analysis Including a New 2D Mapping of Seven‐Membered Rings

**DOI:** 10.1002/cplu.70192

**Published:** 2026-06-14

**Authors:** Filippo Protti, Lucio Toma, Giuseppe Zanoni, Emanuele Casali

**Affiliations:** ^1^ Department of Chemistry University of Pavia Pavia Italy

**Keywords:** 7‐membered rings, code, conformers, cycles, puckering

## Abstract

Ring puckering is a central conformational phenomenon that strongly influences the structure and reactivity of cyclic and heterocyclic molecules, with particular relevance to carbohydrate chemistry and stereoelectronic effects. Ring puckering represents a critical conformational phenomenon in organic chemistry, where cyclic molecules deviate from planarity through out‐of‐plane bending motions to minimize steric and electronic strain. To make quantitative puckering analysis more accessible, this work presents a free, open‐source Python tool that computes Cremer–Pople puckering parameters for five‐, six‐ and seven‐membered rings from standard .xyz coordinates and produces intuitive two‐dimensional visualizations of amplitude and phase. The implementation centers on vectorized projections onto sinusoidal basis functions, special‐case handling for different ring sizes, and interactive selection of ring atoms; it emphasizes clarity, speed, and explicit support for seven‐membered systems often neglected due to the difficulty to represent toroidal landscape. Validation and discussion illustrate how the tool maps canonical envelope, twist, chair, boat, and higher‐dimensional pseudorotation landscapes onto readable plots, enabling rapid classification of conformers and interpretation of dynamical motifs. The program provides a practical and accessible implementation for the analysis and visualization of ring conformations based on puckering coordinates.

## Graphical abstract

CALPUCK is a free, open‐source Python tool for Cremer–Pople puckering analysis of five‐, six‐, and seven‐membered rings. It introduces a novel two‐dimensional mapping for seven‐membered systems, enabling intuitive visualization of their complex conformational landscapes and facilitating rapid classification of ring conformers.

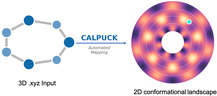

## 1 Introduction

Ring puckering represents a critical conformational phenomenon in organic chemistry, where cyclic molecules deviate from planarity through out‐of‐plane bending motions to minimize steric and electronic strain [1]. This nonplanar distortion is particularly pronounced in saturated and heterocyclic rings, with five‐ and six‐membered systems exhibiting well‐defined puckering patterns that significantly influence molecular properties [2]. In carbohydrate chemistry, the conformational flexibility of furanoid and pyranoid rings directly governs their biological activity, enzymatic recognition, and interactions with host receptors [3, 4, 5, 6]. The precise quantification of ring puckering is essential for understanding stereoelectronic effects, conformational dynamics, and intermolecular interactions in complex molecular systems [5, 6]. The mathematical description of ring puckering has been extensively studied [7]. Traditional displacement‐based approaches to quantifying puckering in five‐, six‐, and seven‐membered rings require specification of a reference plane (*z* = 0) and often involve complex parameterizations [7, 8, 9]. Despite the established theoretical frameworks, computational implementation of puckering analysis remains constrained by the availability and accessibility of computational tools. Recent methodological developments have significantly advanced our capacity to explore ring conformational spaces with greater accuracy and efficiency. In the context of enzymatic catalysis, conformational analyses along reaction coordinates have demonstrated that glycosidases exploit specific substrate puckering pathways such as the transition from chair to half‐chair or envelope conformations to stabilize high‐energy transition states [10]. These insights underscore the importance of accurately characterizing puckering parameters not only for static structural analysis but also for modeling dynamic processes in biochemical systems. Further progress has been made in extending puckering formalisms to larger and more flexible ring systems. For seven‐membered rings, which exhibit richer conformational landscapes due to increased degrees of freedom, Sağıroğlugil et al. introduced extended puckering collective variables specifically designed for enhanced sampling in metadynamics simulations [11]. Their approach enables systematic exploration of complex energy surfaces, revealing previously inaccessible conformational minima and transition pathways that are critical for understanding the behavior of medium‐sized heterocycles in pharmaceutical and materials contexts. Complementing these sampling advances, Lema‐Saavedra and Fernández‐Ramos proposed a general, mathematically rigorous method for bidirectional conversion between Cremer–Pople puckering coordinates and three‐dimensional molecular geometries [12]. This framework provides a robust foundation for conformational search algorithms, allowing researchers to generate physically realistic ring structures directly from puckering parameters or, conversely, to extract meaningful puckering descriptors from optimized geometries, a capability particularly valuable for high‐throughput screening and machine learning applications.

On the software development front, specialized tools have emerged to address niche aspects of ring conformational analysis. For example, MonteCarbo offers an integrated platform for generating and docking multifunctionalized ring molecules, combining conformational sampling with molecular docking protocols tailored for carbohydrate‐inspired systems [13]. While such tools demonstrate the practical utility of puckering‐aware workflows, they often remain focused on specific chemical classes or computational paradigms. More broadly, commercial software packages frequently impose barriers to adoption, including proprietary input formats, steep learning curves, and institutional licensing requirements, which limit their accessibility to researchers in resource‐constrained settings or interdisciplinary teams.

To address these challenges, we present CALPUCK, an open‐source and user‐friendly implementation for the analysis of ring conformations based on established puckering formalisms, featuring integrated visualization tools to facilitate interpretation and application. The program accepts standard .xyz files containing atomic coordinates for generating Cremer–Pople parameters and a clear visualization of ring conformations through custom‐designed graphical representations. This approach provides researchers with an accessible method for analyzing ring puckering without requiring specialized input formats or training. The generated visualizations facilitate intuitive interpretation of puckering amplitudes, phase angles, and ring conformational relationships, offering immediate insights into stereoelectronic effects and molecular interactions relevant to drug design, materials science, and biochemical applications.

## 2 Results and Discussion

### 2.1 Five‐Membered ring

Five‐membered rings display a well‐defined conformational behavior driven by puckering. Five‐membered rings adopt a limited set of nonplanar shapes that are most commonly described as envelope (*E*) and twist (*T*) forms. In an envelope conformation, four atoms lie approximately in a plane while the fifth atom is displaced out of that plane; in a twist conformation, two adjacent atoms are displaced in opposite directions out of the mean plane, producing a slight helical distortion. These discrete labels are convenient snapshots along a continuous pseudorotation pathway: the ring can interconvert smoothly between different *E* and *T* geometries by progressive shifts of which atom is out of plane so that what appear as distinct conformers are actually points on a single low‐energy ring‐puckering manifold [2, 4].

The quantitative description of these shapes uses Cremer–Pople puckering coordinates, which for a five‐membered ring reduce to a total puckering amplitude $q_{2}$ and a phase (pseudorotation) angle $\varphi_{2}$. The amplitude $\;q_{2}$ measures how far atoms deviate from the mean plane (how “puckered” the ring is), while $\varphi_{2}$ locates the ring on the pseudorotation circle and thus identifies whether the instantaneous geometry is closer to an *E* or a *T* form and which atom is the flap or twist center. Mapping computed structures onto the ($q_{2}$, $\varphi_{2}$) plane makes it straightforward to classify conformations, follow pseudorotation trajectories, and compare energy surfaces; this is the basis for most modern puckering analyses and for visualization tools that report both amplitude and phase to the user [2, 4].

**FIGURE 1:**
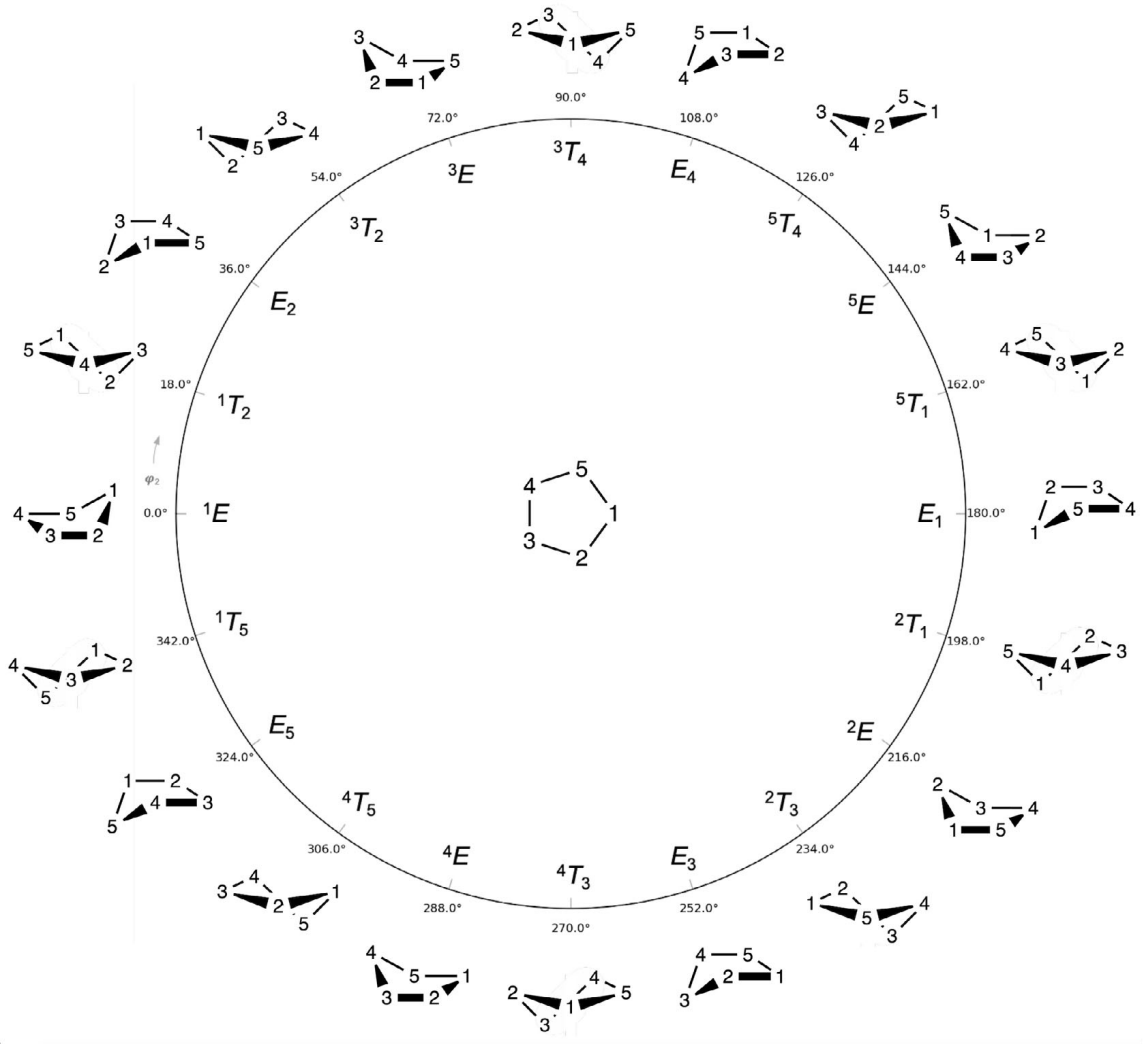
Full conformational landscape for five‐membered rings.

A clear and agnostic representation of a full pseudorotation cycle at a fixed $q_{2}$ for a general five‐membered ring is reported in Figure 1. Around the circle are plotted the discrete snapshots that are most commonly used to label instantaneous geometries: 10 *E* and 10 *T* forms, each corresponding to a different choice of apex atom (for *E*) or twist bond (for *T*). The phase angle $\varphi_{2}$ locates each snapshot on the pseudorotation coordinate and therefore identifies which atom is displaced and whether the form is an original or its inverted counterpart (values differing by $180^{{}^{\circ}}$ are original/inverted pairs) [14].

The schematic adopts the conventional notation used in puckering analyses: a superscript before an *E* denotes that the apex atom is above the mean plane, a subscript after *E* denotes it is below the plane; for *T* forms a superscript before and a subscript after *T* indicate the two atoms of the bond with the largest dihedral angle, with the first atom above and the second below the mean plane. The 20 labeled forms are representative points on a continuous, two‐dimensional conformational manifold (see Figure 1).

### 2.2 Six‐Membered ring

For six‐membered rings, the puckering space is three‐dimensional. These are most conveniently expressed in the Cremer–Pople spherical representation as a total puckering amplitude *Q* and two angles *θ* and $\varphi_{2}$, where *θ* controls the overall type of deformation and therefore distinguishes chair (*θ* ≈ 0° or 180°) from boat and twist‐boat forms, while $\varphi_{2}$ parameterizes the pseudorotational position around the ring and selects specific boat or twist‐boat geometries. This compact triplet therefore maps the familiar discrete names onto continuous coordinates [1, 5].

The three coordinates also make clear the two common dynamical motifs of six‐membered rings. One motif is inversion between the two enantiomeric chairs, which corresponds to motion along the polar axis of the Cremer–Pople sphere and is dominated by changes in *θ* and *Q*. The other motif is pseudorotation among boat and twist‐boat conformers, which appears as motion primarily in $\varphi_{2}$ at roughly constant *θ* and *Q*, allowing to classify minima and transition paths and plot free‐energy surfaces on the (*θ*, $\varphi_{2}$) sphere [5, 15, 16].

These conformational itineraries are not merely geometric abstractions; they are functionally decisive in biological catalysis. As comprehensively demonstrated for glycosidase mechanisms, enzymes actively steer substrate rings through specific puckering pathways, traversing high‐energy boat or skew‐boat regions, to achieve optimal orbital alignment and transition‐state stabilization [10]. Accurately assigning the proper conformational classification requires a representation that preserves both topological continuity and intuitive spatial mapping.

**FIGURE 2:**
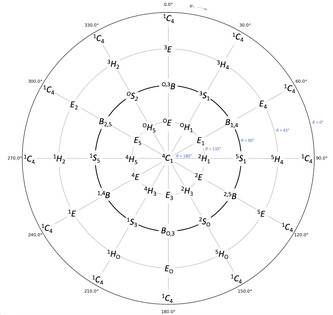
Full conformational 3D landscape for six‐membered rings here flattened into a disk.

**FIGURE 3:**
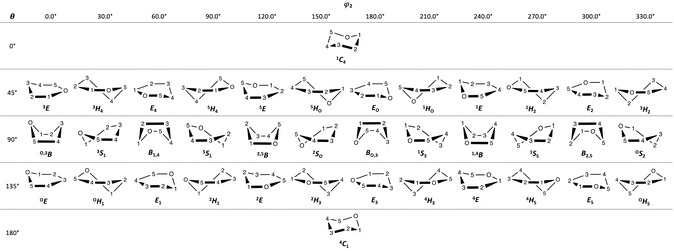
Representations of the conformers for a pyranoside ring.

To simplify the three‐dimensional Cremer–Pople sphere, we propose a bidimensional map, which flattens into a disk made of four concentric rings plus a central point. A closely related representation was reported by Haasnoot in 1992 [17]. The present approach differs in the choice of projection: while Haasnoot's mapping opens the sphere at the pole corresponding to the ^4^ *C*_1_ conformation, in our case the sphere is opened at the opposite pole, corresponding to the ^1^ *C*_4_ conformation. This choice was made to improve the readability of the conformational space in the region most relevant to the systems considered here, by avoiding excessive compression of populated conformations near the projection center and instead distributing them more evenly across the map. Radial position encodes the polar coordinate *θ*: the center corresponds to *θ* **=** 180°, the first ring to *θ* **=** 135°, the second to *θ* **=** 90°, the third to *θ* **=** 45°, and the outermost ring to *θ* **=** 0°. Azimuthal position along each ring encodes the angular coordinate $\varphi_{2}$, so motion around a ring corresponds to changing $\varphi_{2}$ while keeping *θ* fixed. In this layout the canonical chair at one extreme of the polar axis appears at the center, while the opposite chair appears on the outermost ring for every $\varphi_{2}$; intermediate rings host boat and twist‐boat families at the $\varphi_{2}$ positions that correspond to their pseudorotational locations (see Figures 2 and 3).

This representation makes common conformational motions visually intuitive: inversion between chairs appears primarily as radial trajectories across rings, whereas pseudorotation among boats and twist‐boats appears as angular motion around a given ring.

### 2.3 Seven‐Membered ring

A nonplanar seven‐membered ring indeed has four independent out‐of‐plane puckering coordinates, which are commonly expressed as a set of four Cremer–Pople‐style amplitudes and their associated phase angles when a polar decomposition is used: the total puckering amplitude *Q* and three angles *θ*, $\varphi_{2}$ and $\varphi_{3}$. *θ* again acts controlling the relative contributions of the boat‐like and chair‐like conformations, while $\varphi_{2}$ and $\varphi_{3}$ identify one of the multiple equivalent conformations and quantify the relative contributions of the twisted and bent forms. The four coordinates form a four‐dimensional puckering vector that can be decomposed into different 2D subspaces for visualization or analysis. However, the choice of subspaces (and the number of meaningful pseudorotation angles) depends on the chosen basis and on whether one projects the full 4D space onto lower‐dimensional manifolds for interpretation [16, 18]. The mathematical complexity of this higher‐dimensional space has recently been rigorously addressed in the context of enhanced sampling. Sağıroğlugil et al. formalized extended puckering collective variables specifically tailored for seven‐membered rings, demonstrating how these generalized coordinates can efficiently drive metadynamics simulations across highly corrugated energy landscapes [11]. Their work highlights that the additional degrees of freedom in seven‐membered systems require robust coordinate definitions to accurately capture the delicate balance and interconversion between chair, boat, twist‐boat, and skew‐boat families. By successfully mapping these extended variables to free energy surfaces, their study further underscores the necessity of clear, static structural assignment tools to unambiguously classify individual geometries within this multidimensional conformational space. The conformational energy landscape of a seven‐membered ring is therefore four‐dimensional, and while particular high‐symmetry conformers correspond to specific directions in that space, those direction vectors are not generally mutually orthogonal in the mathematical sense unless one constructs an orthonormal basis by explicit linear algebra. Usually, the seven‐ring conformations are mapped onto toroidal or other 2D surfaces by combining pairs of phase angles to produce an intuitive pseudorotation map; such mappings are projections that help visualize minima and pseudorotational pathways but necessarily lose information present in the full 4D description [16, 18, 19].

**FIGURE 4:**
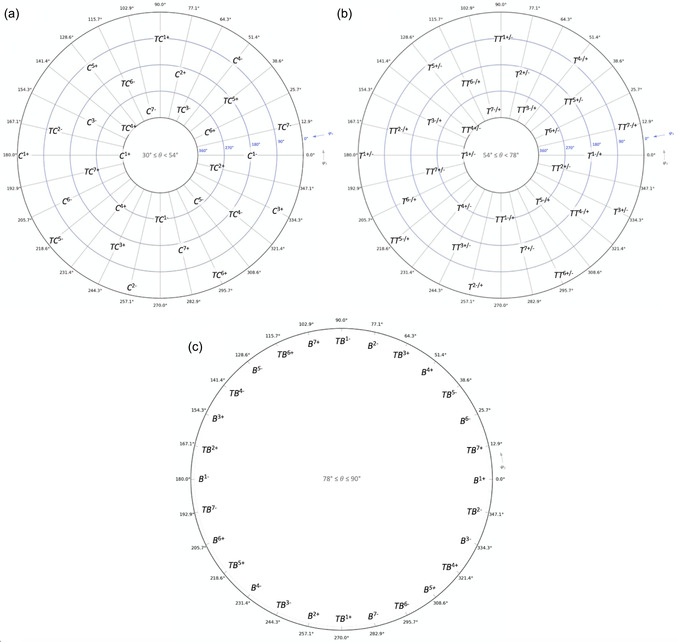
Full conformational landscape for seven‐membered rings. The classical 3D torus is here opened and flattened into three different disks, according to the θ values: (a) 30° ≤ *θ* < 54°; (b) 54° ≤ *θ* < 78° and (c) 78° ≤ *θ* ≤ 90°.

In the present work, we introduce a 2D map representation that offers an alternative way to visualize the underlying toroidal topology. Here, the angular coordinate around the plot is assigned to $\varphi_{2}$ while the radial (concentric) coordinate encodes $\varphi_{3}$. The angular axis is sampled in 12.9° steps so that successive points along a given concentric circle represent incremental changes in $\varphi_{2}$. The radial axis representing $\varphi_{3}$ is sampled in 90° steps. Reading the diagram, therefore, amounts to reading a two‐angle projection of the full four‐dimensional puckering vector: the azimuthal position gives the $\varphi_{2}$ phase, and the ring index gives the $\varphi_{3}$ phase, while the plotted spiral encodes how the two phases combine for a fixed value of the polar mixing angle *θ* (see Figure 4).

Because the torus has been opened after swapping the roles of $\varphi_{2}$ and $\varphi_{3}$, the plotted spiral is continuous, and the innermost point labeled coincides with the outermost point. This topological identification is the visual manifestation of the toroidal periodicity: moving radially inward by one full set of $\varphi_{3}$ steps and simultaneously advancing azimuthally by the appropriate number of $\varphi_{2}$ steps returns the system to the same puckering state. In practice, this means that the spiral is a convenient way to display the pseudorotational equivalence classes while keeping the grid readable.

**FIGURE 5:**
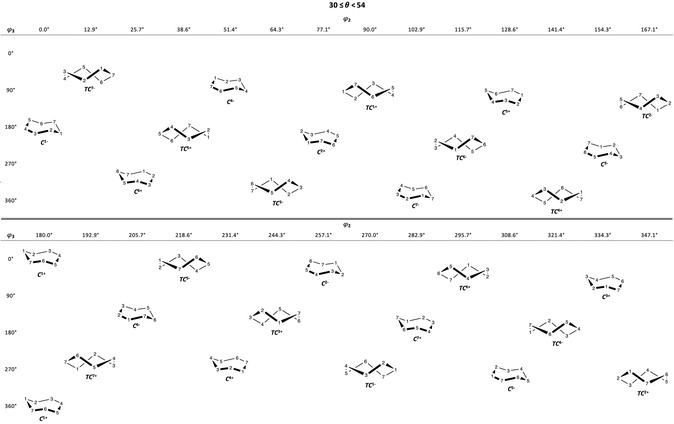
Seven‐membered ring representations of the conformers for 30 ≤ θ < 54.

**FIGURE 6:**
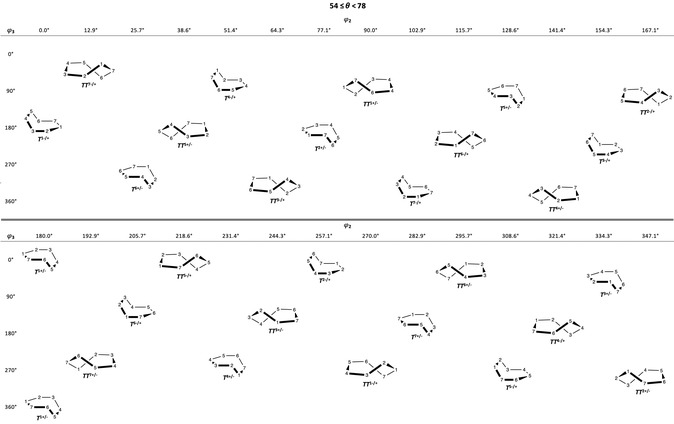
Seven‐membered ring representations of the conformers for 54 ≤ θ < 78.

**FIGURE 7:**
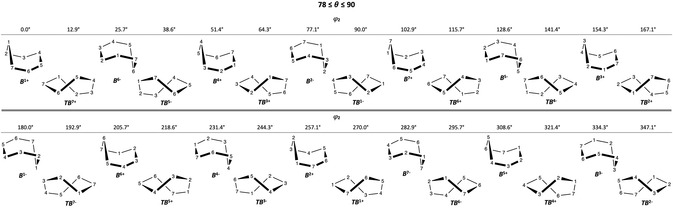
7‐membered ring representations of the conformers for 78 ≤ θ ≤ 90.

The distribution of conformers along the spiral depends strongly on the mixing angle θ that controls the relative weight of the two 2D subspaces. For θ between 30° and 54°, the map shows one family of minima and connecting pathways characteristic of partially chair‐like (*C*) and twisted‐chair (*TC*) conformations (Figure 3a). For θ between 54° and 78°, a different set of conformations appears. Within this range of values, the transition states (*T*) between boats (*B*) and chairs (*C*) and the twisted‐transition states (*TT*) between twisted‐boats (*TB*) and twisted‐chairs (*TC*) are represented (Figure 3b) [18]. For θ between 78° and 90°, the landscape collapses to a near‐one‐dimensional ring of conformations that vary primarily with $\varphi_{2}$ (Figure 3c) [19]. It represents a group of minima and connecting pathways between boat‐like (*B*) and twisted‐boats (*TB*). The sketch representations of the conformers for a seven‐membered ring are reported in Figures 5, 6–7 accordingly with the combinations of θ, $\varphi_{2}$, and $\varphi_{3}$. It should be emphasized that the proposed 2D representation is a projection of the underlying higher‐dimensional conformational space. As such, it is primarily intended to facilitate visualization and qualitative interpretation and does not constitute a general or exhaustive description of seven‐membered ring conformations.

### 2.4 Code Overview

The program is designed as a compact, practical tool that translates Cartesian coordinates from an .xyz file into the scalar and angular descriptors commonly used to classify ring conformations. At its core, the program implements the standard Cremer–Pople formalism: coordinates are centered, atomic displacements are projected onto orthogonal sinusoidal basis functions, and the resulting mode amplitudes are combined to yield the *q* components, the total puckering amplitude *Q*, and the spherical angles θ and φ that define the ring conformation. The implementation uses vectorized operations to compute the sine‐ and cosine‐weighted projections and the normal to the mean plane, which keeps the numerical flow concise and efficient. Special‐case handling is included for the different algebraic forms required by five–, six‐, and seven‐membered rings.

**FIGURE 8:**
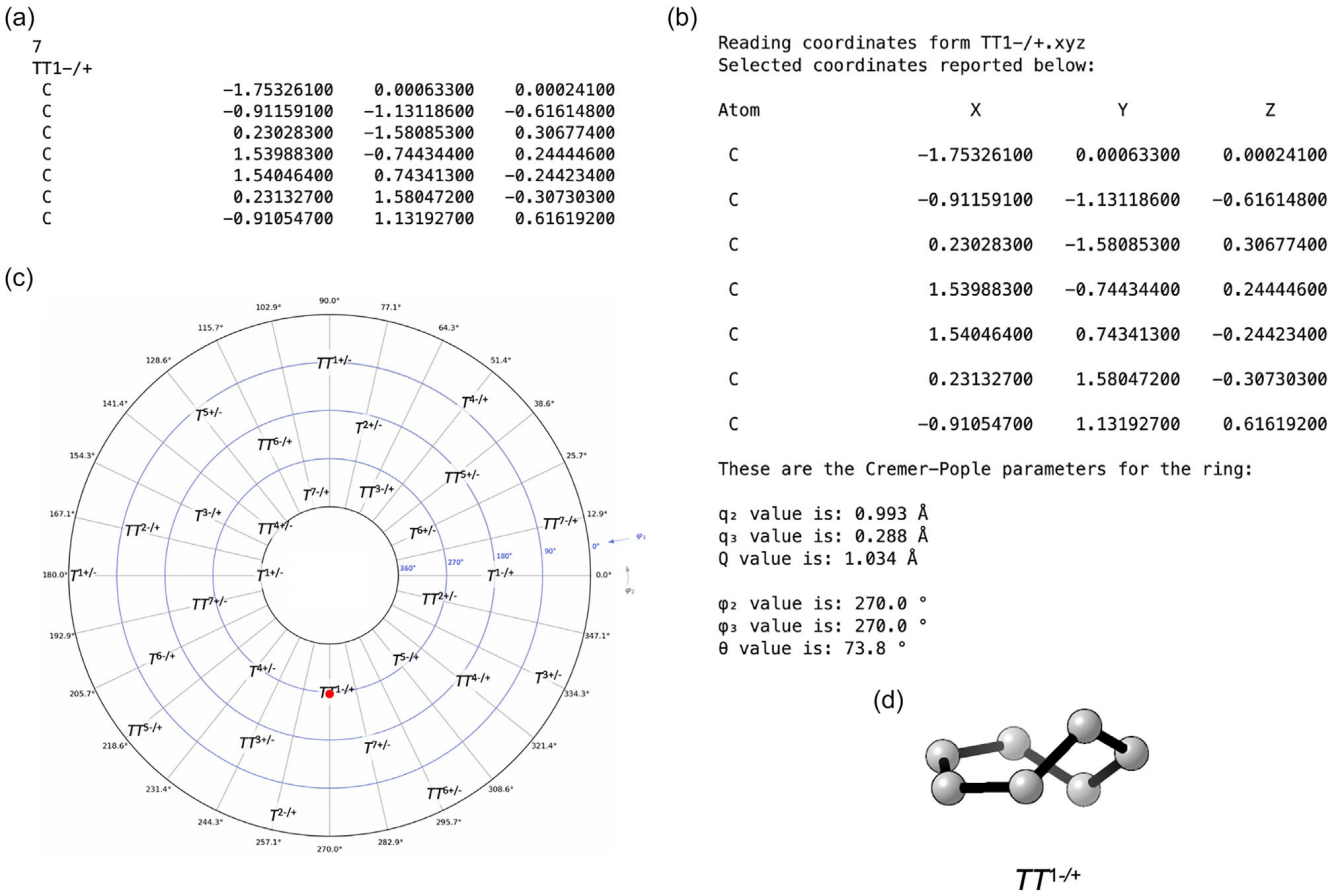
Example of the code running on the twisted‐transition state *TT* ^*1−/+*^ conformation in a cycloheptane ring interconversion between *TC* ^*1−*^ and *TB* ^*1+*^. (a) Cartesian coordinates for *TT* ^*1−/+*^ conformation; (b) Code output with puckering parameters; (c) Visual representation output for the *TT* ^*1−/+*^ conformation within the seven‐membered ring conformational landscape; (d) 3D representation of the *TT* ^*1−/+*^ conformation.

The program relies on a short interactive prompt to identify which lines in the file correspond to the ring atoms (Figure 8a). This design choice keeps the code simple and flexible for manual use, but it also implies that the user must supply the correct atom indices at runtime. Selected coordinates are echoed to the terminal for verification, and the final numerical results are printed in a human‐readable format with standard units (Figure 8b).

Output is intentionally immediate and visual thanks to the fact that, in addition to the numeric Cremer–Pople parameters, the script produces a two‐dimensional graphical marker on preprepared scheme images that represent the puckering map for the given ring size (Figure 8c).

Some practical features make this implementation particularly useful in a research setting. Its open‐source nature means the code is concise, readable and available for inspection and modification. This contrasts with many commercial packages that perform similar analyses behind closed doors. Moreover, the program explicitly supports seven‐membered rings, a capability that is uncommon in off‐the‐shelf tools. The computational cost for a single ring is negligible, and the program returns results essentially instantaneously on modern hardware.

In its current implementation, CALPUCK is primarily designed for interactive analysis of individual structures, where ring atoms are explicitly defined by the user to ensure robustness across chemically diverse systems. While limited batch processing is possible for sets of structures with consistent atom indexing, automated trajectory‐scale analysis is not presently supported.

## 3 Conclusion

This program provides a pragmatic, open, and extensible implementation of Cremer–Pople puckering analysis for five‐, six‐, and seven‐membered rings. Its principal strengths are clarity, computational efficiency, and the introduction of a bidimensional conformational mapping framework for seven‐membered rings within an open‐source environment. While five‐ and six‐membered ring puckering representations are well established, seven‐membered systems remain significantly less standardized due to their higher‐dimensional conformational space and intrinsic flexibility. The present implementation addresses this gap by providing a reduced, yet information‐rich, two‐dimensional mapping that enables intuitive visualization and systematic comparison of seven‐membered ring conformations. This bidimensional representation constitutes a practical conceptual advance, transforming a formally complex conformational landscape into an accessible analytical tool without loss of essential structural information.

The current design favors immediate, interactive use and clear visual feedback, while leaving ample room for future extensions, including automated workflows, expanded file format compatibility, and platform‐independent visualization. For researchers requiring a transparent and modifiable framework to compute and analyze puckering parameters, this implementation provides a transparent and modifiable framework for computing and analyzing puckering parameters, facilitating their application in computational studies.

## Funding

This study was supported by Regione Lombardia (BRAVE (PR FESR 2021‐2027)).

## Conflicts of Interest

The authors declare no conflicts of interest.

## Data Availability

The data that support the findings of this study are openly available in CALPUCK at https://github.com/emacasali/CALPUCK.
